# Real-world experience quantifying access to JAK inhibitor care for alopecia areata patients: a patient-centered survey study

**DOI:** 10.1097/JW9.0000000000000145

**Published:** 2024-04-11

**Authors:** Ambika Nohria, Ya-Han (Crystal) Zhang, Deesha Desai, Alison Lee, Lisa Anderson, Jerry Shapiro, Maryanne Senna, Kristen Lo Sicco

**Affiliations:** a The Ronald O. Perelman Department of Dermatology, New York University Grossman School of Medicine, New York, New York; b University of Pittsburgh School of Medicine, Pittsburgh, Pennsylvania; c The Spence School, New York, New York; d National Alopecia Areata Foundation, San Rafael, California; e Lahey Hair Loss Center of Excellence and Research Unit, Burlington, Massachusetts

**Keywords:** access to care, alopecia areata, Janus kinase (JAK) inhibitor

## Abstract

**Background:**

Janus kinase inhibitor (JAKi) therapy has revolutionized the treatment landscape for alopecia areata (AA); however, access may be limited by a lack of insurance coverage and high out-of-pocket costs.

**Objective:**

We aimed to evaluate real-world patient experiences regarding access to JAKi therapy.

**Methods:**

We conducted an online patient-centered survey using the National Alopecia Areata Foundation listserv.

**Results:**

In total 784 individuals initiated our survey, and 600 completed it in full (76.5%). While more non-White patients considered obtaining JAKi therapy, more White patients reported the use of this medication class. In total, 74.2% lacked insurance coverage or had partial coverage for JAKi, and 52% expressed dissatisfaction with available coverage. However, 52.9% reported delays in starting medication due to insurance approval processes, contributing to worsened AA and related stress. In total, 35% of patients did not try to obtain JAKi therapy due to concerns about costs, and 18.2% discontinued therapy due to financial barriers. Also, 19.8% of patients reported utilizing financial savings to pay for medication, and 55.2% reported using a copay assistance card. Further, 12.2% reported forgoing other necessities to pay for AA expenses.

**Limitations:**

Our results are limited by the subjective nature of survey studies. The recency of FDA approval for JAKi therapy may also influence patients’ perceptions of access to care.

**Conclusion:**

Patients with AA face significant barriers when trying to obtain JAKi therapy, and existing racial inequities may be exacerbated by these barriers. Further advocacy work is needed to improve access to care.

**What is known about this subject in regard to women and their families?**

- Alopecia areata (AA) disproportionately affects women globally, presenting both physical and psychosocial challenges.
- Accessing and affording AA treatment poses difficulties for all patients, with women, especially those supporting children, facing amplified challenges as primary emotional and financial caregivers.

**What is new from this article as messages for women and their families?**

- Our study shows that women, who make up the majority of respondents (80.2%), are significantly affected by the challenges associated with AA.
- Our findings reveal that many patients, including women, face difficulties in obtaining insurance coverage for treatment, leading to delays and financial burdens.
- Overall, the study emphasizes the significant barriers that all patients encounter while seeking treatment for AA.

## Introduction

Alopecia areata (AA) is an autoimmune disease characterized by nonscarring hair loss that can affect any hair-bearing site on the body. AA can have a significant negative impact on quality of life and be a source of psychosocial distress.^1^

Treatment of AA has traditionally involved off-label use of corticosteroids, topical and low-dose oral minoxidil, and systemic immunotherapies among other agents. However, in June 2022 the United States Food and Drug Administration (FDA) approved the first oral Janus kinase inhibitor (JAKi), baricitinib, for adults with severe AA.^2^ Shortly thereafter, in June 2023, the FDA approved ritlecitinib, the first oral JAKi approved for ages 12 and older.^3^

While oral JAKi offers hope for more efficacious AA treatment, access to these medications may be limited by high medication costs and insurance coverage difficulties. A 2020 study evaluating access to the JAKi tofacitinib, which was prescribed off-label for the treatment of AA, found that 31% of patients received no insurance coverage, and those who managed to obtain coverage often required one or more appeals before receiving a final decision.^4^

To our knowledge, no similar studies have been conducted to quantify access to oral JAKi in the setting of recent FDA approval for AA. Toward this effort, we conducted a patient-centered survey study to quantify real-world experience regarding JAKi therapy, including an assessment of potential barriers to accessing care.

## Methods

A Web-based RedCap survey was using the National Alopecia Areata Foundation’s (NAAF) listserv from September 11, 2023 to October 19, 2023. Adults and parents of patients under 18 were surveyed. This study was approved by the NYU Langone Health Institutional Review Board. *χ*^2^ tests were used to assess associations. *P* values <.05 were deemed statistically significant.

## Results

A total of 784 individuals initiated the survey, with 600 surveys completed in full (76.5%). Those who did not complete the survey in full were excluded from our analysis (23.5%). Most respondents were women (80.2% female, 19.8% male), and White (73.8%), with a mean (SD) age of 43 (21) years. However, 70.7% of patients reported >50% scalp involvement (Table 1).

**Table 1:** Patient characteristics and quality of life assessment

Survey completion	N = 600
Sex	
Female, *n*% Male, *n*% Nonbinary, *n*% Prefer not to disclose, *n*%	481 (80.2%) 117 (19.8%) 1 (0.2%) 1 (0.2%)
Age Mean (range) Under 18 years old, *n*% Over 18 years old, *n*%	43 (2.5-85) 122 (20.3%) 478 (79.7%)
Race/ethnicity	
White, *n*% Non-White *n*%	443 (73.8%) 157 (26.2%)
Region of residence	
Northeast, *n*% Southeast, *n*% Midwest, *n*% Southwest, *n*% West, *n*%	137 (22.8%) 150 (25%) 143 (23.8%) 53 (8.8%) 117 (19.5%)
% Scalp involvement	
<20%, *n*% 20-50%, *n*% >50%, *n*%	83 (13.8%) 93 (15.5%) 424 (70.7%)
Duration of current hair loss	
<1 year, *n*% 1-5 years, *n*% 5-10 years, *n*% 10-20 years, *n*% 20+ years, *n*%	33 (5.5%) 175 (29.2%) 126 (21%) 102 (17%) 164 (27.3%)
How often bothered by AA in the past week?	
Never, *n*% Sometimes, *n*% Often, *n*% Always, *n*% >50% scalp involvement Never, *n*% Sometimes, *n*% Often, *n*% Always, *n*% ≤50% scalp involvement Never, *n*% Sometimes, *n*% Often, *n*% Always, *n*%	100 (16.7%) 231 (38.5%) 119 (19.8%) 150 (25%) 71 (16.7%) 160 (37.7%) 84 (19.8%) 109 (25.7%) 29 (16.5%) 71 (40.3%) 35 (19.9%) 41 (23.3%)
How often emotionally bothered by AA in the past week?	
Never, *n*% Sometimes, *n*% Often, *n*% Always, *n*% >50% scalp involvement Never, *n*% Sometimes, *n*% Often, *n*% Always, *n*% ≤50% scalp involvement Never, *n*% Sometimes, *n*% Often, *n*% Always, *n*%	89 (14.8%) 244 (40.7%) 138 (23%) 129 (21.5%) 64 (15.1%) 170 (41.1%) 94 (22.2%) 96 (22.6%) 25 (14.2%) 74 (42.0%) 44 (25.0%) 25 (18.8%)
How often bothered by impact of AA on activities in the past week?	
Never, *n*% Sometimes Often Always >50% scalp involvement Never, *n*%	172 (28.7%) 223 (37.2%) 117 (19.5%) 88 (14.7%) 115 (27.1%)
Sometimes, *n*% Often, *n*% Always, *n*% ≤0% scalp involvement Never, *n*% Sometimes, *n*% Often, *n*% Always, *n*%	150 (35.4%) 85 (20.0%) 74 (17.5%) 57 (32.4%) 73 (41.5%) 32 (18.2%) 14 (8.0%)

AA, alopecia areata.

We evaluated the impact of AA on quality of life across multiple domains. When asked how often AA bothered them overall, for our overall cohort, 16.7% of patients reported never, 58.3% sometimes or often, and 25% always. When asked how often AA bothered them emotionally, 14.8% of patients reported never, 63.7% sometimes or often, and 21.5% always. When asked how often AA impacted their activities, 28.7% of patients reported never, 56.7% sometimes or often, and 14.7% always. We compared quality of life measures between patients who had >50% scalp involvement and those with 50% or less scalp involvement. When asked how often AA bothered them overall, patients with >50% scalp involvement reported 16.7% never, 57.8% sometimes or often, and 25.7% always compared to those with ≤50% scalp involvement who reported 16.5% never, 60.2% sometimes or often, and 23.3% always. When asked how often AA bothered them emotionally, for those with >50% scalp involvement, 15.1% reported never, 62.5% sometimes or often, and 22.6% always compared to those with ≤50% scalp involvement who reported 14.2% never, 67.6% sometimes or often, and 18.8% never. When asked how often AA impacted their activities, for those with >50% scalp involvement, 27.1% reported never, 55.7% sometimes or often, and 17.5% always compared to those with ≤50% scalp involvement who reported 32.4% never, 60.2% sometimes or often, and 8% always (Table 1).

Within our cohort, 58.7% of patients were not currently receiving any form of medical treatment for their AA, including 65.3% of patients with >50% scalp involvement and 42.6% of patients with ≤50% scalp involvement. Of these patients, 13.4% endorsed wanting to start treatment but being unable to get insurance coverage and 20.7% endorsed not starting treatment due to cost concerns.

When asked if they had considered obtaining treatment with JAKi specifically, regardless of whether they took action to obtain medication, 56.1% reported yes, with significantly more non-White patients reporting considering obtaining therapy (*P* = .000014). Beyond just consideration, when asked about whether they had taken steps to try and obtain JAKi, 37.7% of patients reported yes, with significantly more White participants and patients with >50% scalp involvement trying to obtain therapy (*P* = .033, .0071, respectively). Among patients that did not try to obtain JAKi, reasons for not pursuing this therapy included concerns about side effects for 50.8%, concerns about cost for 35%, and 14.2% did not try to obtain therapy as they reported not being bothered by their hair loss.

Also, 28.7% of patients reported they are currently or had previously taken JAKi therapy with significantly more White participants and patients with >50% scalp involvement using JAKi (*P* = .0020, .0022, respectively). Of these patients, 45.3% endorsed receiving JAKi off-label for their AA, for example, oral tofacitinib or topical ruxolitinib, which are not FDA-approved for the treatment of AA. About 89.5% and 28.5% reported the use of oral and topical JAKi, respectively. A total of 33 patients reported previously taking JAKi therapy but discontinuing the use of the medication. Of these patients, 30.3% reported intolerable side effects, 9.1% reported paying out of pocket and no longer being able to afford the medication, 9.1% reported use of free samples which they were no longer able to access, and 51.5% cited other reasons for discontinuing therapy (Table 2).

**Table 2:** Patient use of JAKi therapy

Survey completion	N = 600
Currently receiving any medical therapy	N = 600
Yes, *n*% No, *n*%	248 (41.3%) 352 (58.7%)
Reason for not currently receiving medical therapy	N = 352
I choose to forego medical treatment and feel comfortable not wearing a scalp covering, *n*% I choose to forego medical treatment and wear a wig or topper when in public, *n*% I choose to forego medical treatment and wear a cap/hat/bandana when in public, *n*% I wanted to start medical treatment for my alopecia areata, but could not get it covered by my insurance company, *n*% I previously received medical treatment that was not successful and not pursued further treatment(s), *n*% I have not started medical treatment because of concerns about side effects, *n*% I have not started medical treatment because of concerns about costs, *n*%	50 (14.2%) 89 (25.3%) 45 (12.8%) 47 (13.4%) 227 (64.5%) 85 (24.1%) 73 (20.7%)
Considered taking JAKi therapy	N = 600
Yes I have considered it and a doctor has recommended a JAKi to treat my/my child’s alopecia areata, *n*% Yes I have considered it but a doctor has not recommended a JAKi to treat my/my child’s alopecia areata, *n*% No I have not considered it but a doctor has recommended a JAKi to treat my/my child’s alopecia areata, *n*% No I have not considered it and a doctor has not recommended a JAKi to treat my/my child’s alopecia areata, *n*%	229 (38.2%) 108 (18%) 24 (4%) 239 (39.8%)
Tried to obtain JAKi therapy	N = 600
Yes, *n*% No, *n*%	226 (37.7%) 374 (62.3%)
Reason for not trying to obtain JAKi therapy	N = 374
Concerns about side effects, *n*% Concerns about cost, *n*% Not bothered by hair loss, *n*%	190 (50.8%) 131 (35%) 53 (14.2%)
Taking JAKi therapy	N = 600
Yes, currently taking JAKi, *n*% Yes, but no longer taking JAKi, *n*% No, never taken *n*%	139 (21.7%) 33 (5.5%) 428 (71.3%)
Reason for stopping JAKi	N = 33
Was taking samples because insurance did not cover the medication and could no longer access samples, *n*% Was paying out of pocket and could no longer afford it, *n*% Had side effects that were not tolerated, *n*% Other, *n*%	3 (9.1%) 3 (9.1%) 10 (30.3%) 17 (51.5%)
Use of topical JAKi	N = 172
Yes, *n*% No, *n*%	49 (28.5%) 123 (71.5%)
Use of oral JAKi	
Yes, *n*% No, *n*%	154 (89.5%) 18 (10.5%)
Specific oral JAKi	N = 154
Xeljanz (Tofacitinib) 5 mg every other day, *n*% Xeljanz (Tofacitinib) 5 mg once daily, *n*% Xeljanz (Tofacitinib) 5 mg twice daily, *n*% Xeljanz (Tofacitinib) 5 mg three times daily, *n*% Olumiant (Baricitinib) 2 mg once daily, *n*% Olumiant (Baricitinib) 4 mg once daily, *n*% Litfulo (Ritlecitinib) 50 mg once daily, *n*% Other (oral ruxolitinib, upadacitinib, deuruxolitinib clinical trial)	4 (2.6%) 11 (7.1%) 28 (18.2%) 5 (3.2%) 26 (16.9%) 62 (40.3%) 10 (6.5%) 12 (7.8%)

JAKi, Janus kinase inhibitor.

Regarding insurance coverage for JAKi, 41.3% reported no coverage, 33.1% had partial coverage, and 25.6% had full coverage. Insurance coverage was significantly less likely to occur for those prescribed JAKi off-label for AA (*P* = .02).

Regarding satisfaction with insurance coverage, 43% reported feeling very inadequate, 11% inadequate, 12.8% neutral, 12.8% adequate, and 20.3% very adequate. Patients were more likely to endorse feeling inadequate satisfaction if they had <50% scalp involvement (*P* = .002).

Regarding medication initiation, 52.9% endorsed experiencing delays due to the insurance approval process. About 49.5% and 69.2% of these patients felt the delay worsened their AA or AA-related stress, respectively, with 22% facing a delay of greater than 6 months (Table 3).

**Table 3:** Insurance coverage and financial considerations for JAKi use

Patients reporting current or prior use of JAKi	N = 172
Insurance coverage of JAKi	N = 172
No coverage, *n*% Partial coverage, *n*% Full coverage, *n*%	71 (41.3%) 57 (33.1%) 44 (25.6)
Satisfaction with insurance coverage for JAKi	N = 172
Very Inadequate, *n*% Inadequate, *n*% Neutral, *n*% Adequate, *n*% Very Adequate, *n*%	74 (43%) 19 (11%) 22 (12.8%) 22 (12.8%) 35 (20.3%)
Delay in care due to insurance	N = 172
Yes, *n*% No, *n*%	91 (52.9%) 81 (47.15%)
Length of delay in care	N = 91
1 month, *n*% 2 months, *n*% 3 months, *n*% 4 months, *n*% 5 months, *n*% >6 months, *n*%	17 (18.7%) 24 (26.4%) 21 (23.1%) 9 (9.9%) 0 (0%) 20 (22%)
Impact of delay in care on AA Worsened AA	N = 91
Yes, *n*% No, *n*%	45 (49.5%) 46 (50.5%)
Worsened AA-related stress	
Yes, *n*% No, *n*%	63 (69.2%) 28 (30.8%)
JAKi on vs off-label	N = 172
Off-label for AA, *n*% On-label for AA, *n*% On-label for a condition other than AA, *n*%	78 (45.3%) 94 (54.7%) 64 (37.2%)
Utilization of copay assistance card	N = 172
Yes, *n*% No, *n*% Not aware of copay assistance card, *n*% Not approved for copay assistance card, *n*%	95 (55.2%) 49 (28.5%) 18 (10.5%) 10 (5.8%)
Utilization of financial savings	N = 172
Yes, *n*% No, *n*%	34 (19.8%) 138 (80.2%)
Foregoing basic necessities or desired items	N = 172
Yes, *n*% No, *n*%	21 (12.2%) 151 (87.8%)
Items given up	N = 21
Meals, *n*% Gas, *n*% Vacation travel, *n*% Gym classes/memberships, *n*% School supplies, *n*% Leisure activities *n*% Other, *n*%	4 (19%) 5 (23.8%) 17 (81%) 9 (42.9%) 1 (4.8%) 19 (90.5%) 2 (9.5%)

AA, alopecia areata; JAKi, Janus kinase inhibitor.

In total 55.2% of patients reported the use of a copay assistance card. White participants and patients receiving treatment on-label for AA were more likely to report using a copay assistance card (*P* = .024, *P* = .005, respectively). About 12.2% of patients reported forgoing necessities to pay for therapy, including meals, gas, vacation/travel, school supplies, and leisure activities. Additionally, 19.8% of patients reported utilizing financial savings to pay for treatment. Patients were significantly more likely to use savings if therapy was received off-label (*P* = .011) (Table 3).

## Discussion

Our study highlights the significant barriers related to cost and insurance coverage that patients with AA face trying to access treatment. This work expands upon previous research identifying the burdens on the AA community, with some reporting the use of savings, cutting down on recreational activities, and even limiting food or clothes to manage expenses.^5,6^ We corroborate these findings, demonstrating that patients report giving up basic necessities, including meals, gas, and school supplies to afford medical therapy. This highlights the profound impact that AA can have on a patient’s life, beyond the disruption to appearance alone. Our results indicate that many patients face challenges obtaining insurance coverage and feel inadequate satisfaction with the coverage received. Furthermore, some report having to utilize financial savings or a copay assistance card, all of which possibly contribute to a delay in accessing medication, which worsens their AA or AA-related stress.

Our study also demonstrates the profound impact of AA on quality of life, with 83% of patients reporting being bothered by AA, 85% reporting being emotionally bothered by AA, and 71% reporting being bothered by the impacts of AA on their activities. Of note, both patients with >50% scalp involvement and those with ≤50% scalp involvement reported similar impacts on quality of life. It is important to recognize that even those with less severe disease are still significantly impacted by AA and may require or desire medical therapy. Unfortunately, most insurance companies require documentation of a severity of alopecia tool score of 50 or higher to provide coverage for JAKi. Our findings highlight the need for expanding coverage, as a quality of life burden is evidently present regardless of baseline severity of alopecia tool score.

Despite significant impacts on quality of life, 58.7% of survey respondents were not currently receiving treatment for their AA. This may in part be due to financial concerns, which not only delay patient care but may also prevent patients from accessing medication altogether. Our findings demonstrate that many patients did not try to obtain JAKi therapy due to concerns about costs, inhibiting them from even trialing this efficacious medication class. Further, numerous patients had to discontinue therapy due to an inability to continue paying out of pocket for medication or a lack of access to free samples which they relied on due to lack of insurance coverage. Our results highlight that patients may not begin or may discontinue beneficial medical therapy purely due to economic barriers.

Beyond medication costs, many patients with AA face additional expenses when trying to obtain camouflaging agents, including cranial prostheses (CP). A 2023 study estimated the average cost of a CP to be $1,543, with only 23% of patients receiving any form of insurance coverage for the piece.^7^ To alleviate this burden, the bill H.R. 3332, *Wigs as Durable Medical Equipment Act*, was introduced to the US House of Representatives in September 2021, calling for wigs to be reclassified as durable medical equipment and be covered by Medicare. Supporting measures like H.R. 3332 (now H.R. 4034 and S. 1922) serve as an initial step toward alleviating some of the significant financial burden placed on patients with AA. Further legislative work is called for to address the high cost and lack of insurance coverage for medication therapy, as highlighted by our work.

Research has shown that systemic racial and socioeconomic inequalities impact access to dermatologic care, and limited medication insurance coverage may exacerbate these inequities.^8^ Our findings demonstrated that while significantly more non-White patients considered obtaining JAKi therapy, significantly more white patients tried to obtain the medication and endorsed currently or previously taking JAKi. These differences may reflect the impact of demographics on medication access.

## Limitations

There are several limitations to our study. Results are retrospective and self-reported; thus, they may not always represent patient experience with high accuracy. Our survey was distributed 14 months after the FDA approval of baricitinib and 2 months after the approval of ritlecitinib. Many patients, particularly pediatric patients, may have obtained their medication before FDA approval, influencing their perceptions of access and financial burden. 45% of patients received their JAKi off-label, which may also affect their experiences.

## Conclusion

As treatment options for patients with AA continue to expand, we hope that this information highlights the barriers this community faces when accessing care. Growing advocacy efforts for broader coverage for treatment and camouflaging agents to alleviate the burden that patients face is essential for ensuring appropriate care for this community.

## Conflicts of interest

The authors made the following disclosures: K.L.S. has been an investigator for Regen Lab and is an investigator for Pfizer. He is a consultant for Pfizer and Aquis. J.S. is an investigator and consultant for Pfizer and is a consultant for Lilly. For other authors, there is no conflicts of interest.

## Funding

None.

## Study approval

The authors confirm that any aspect of the work covered in this manuscript that has involved human patients has been conducted with the ethical approval of all relevant bodies. This study was approved by the NYU Langone Health Institutional Review Board.

## Author contributions

AN, Y-H(C)Z, DD, and AL: Contributed to the conception and writing of this article. LA, JS, MS, and KLS: Contributed to the conception and editing of this article.
